# In-Home Positioning for Remote Home Health Monitoring in Older Adults: Systematic Review

**DOI:** 10.2196/57320

**Published:** 2024-12-02

**Authors:** Andrew Chan, Joanne Cai, Linna Qian, Brendan Coutts, Steven Phan, Geoff Gregson, Michael Lipsett, Adriana M Ríos Rincón

**Affiliations:** 1 Glenrose Rehabilitation Hospital Edmonton, AB Canada; 2 University of Alberta Edmonton, AB Canada

**Keywords:** gerontology, geriatrics, older adult, elderly, aging, aging-in-place, localization, ambient sensor, wearable sensor, acceptability, home monitor, health monitor, technology, digital health, e-health, telehealth, clinical studies, cognitive impairment, neuro, cognition

## Abstract

**Background:**

With the growing proportion of Canadians aged >65 years, smart home and health monitoring technologies may help older adults manage chronic disease and support aging in place. Localization technologies have been used to support the management of frailty and dementia by detecting activities in the home.

**Objective:**

This systematic review aims to summarize the clinical evidence for in-home localization technologies, review the acceptability of monitoring, and summarize the range of technologies being used for in-home localization.

**Methods:**

The PRISMA (Preferred Reporting Items for Systematic Reviews and Meta-Analyses) methodology was followed. MEDLINE, Embase, CINAHL, and Scopus were searched with 2 reviewers performing screening, extractions, and quality assessments.

**Results:**

A total of 1935 articles were found, with 36 technology-focused articles and 10 articles that reported on patient outcomes being included. From moderate- to high-quality studies, 2 studies reported mixed results on identifying mild cognitive dementia or frailty, while 4 studies reported mixed results on the acceptability of localization technology. Technologies included ambient sensors; Bluetooth- or Wi-Fi–received signal strength; localizer tags using radio frequency identification, ultra-wideband, Zigbee, or GPS; and inertial measurement units with localizer tags.

**Conclusions:**

The clinical utility of localization remains mixed, with in-home sensors not being able to differentiate between older adults with healthy cognition and older adults with mild cognitive impairment. However, frailty was detectable using in-home sensors. Acceptability is moderately positive, particularly with ambient sensors. Localization technologies can achieve room detection accuracies up to 92% and linear accuracies of up to 5-20 cm that may be promising for future clinical applications.

**Trial Registration:**

PROSPERO CRD42022339845; https://www.crd.york.ac.uk/prospero/display_record.php?RecordID=339845

## Introduction

The proportion of Canadians aged >65 years is growing, from 7 million in 2020 to an estimated 9.5 million (23% of the population) by 2030 [1-3]. With the ratio of adults aged 15-64 years to persons aged 65 years and older halving from 7.2 in 1980 to 3.6 in 2020, the question of how to maintain a sustainable health care system in the face of these changing demographics remains a top priority [1]. Transforming care processes by using digital platforms and remote monitoring tools may be able to address our increasingly older population and lead to higher life expectancies [4]. Smart home and health monitoring technologies have been touted as the future of managing chronic diseases and allowing people to age in place and live within the comfort and familiarity of their own homes for longer [5-8].

Aging is often accompanied by a gradual decrease in physical and mental capacity [9,10]. In-home monitoring technologies have been used to support older adults to age in place by detecting and managing worsening physical and cognitive decline [7,8,11-19]. Wearables, including accelerometers and gyroscopes, have been used to monitor postural transitions [20] and provide yearly gait speed assessments [21], while weight scales and grip balls have been used to monitor changes in weight and grip strength [22]. Actigraphy has been commonly used in cross-sectional studies on physical activity and gait alongside ambient sensors [13] and to monitor behavioral changes such as agitation and aggression [12,16].

In order to identify appropriate interventions for aging in place, technologies need to first identify body postures and positions that can be reliably interpreted as a functional activity of daily living. While actigraphy can give some quantitative idea of the amount of movement happening, it lacks contextual data that could allow for targeted interventions and improved interpretation of activity data [15,23-25]. Localization technologies are key to providing context that helps with reliable interpretation of what activities are being done. Ambient monitors, including infrared sensors and magnetic door contact sensors, can detect which room a resident is in, determine if they are cooking elaborate or simple dishes, or identify if they are doing self-care activities such as mopping or laundry. This context may be a more sensitive factor in the early detection of dementia, cognitive decline, or increased risk of falls among older adults [26-28]. Wearable tags using wireless technologies such as Bluetooth or Wi-Fi can also be used to localize residents in their homes, offering 1.5-5 m, or room-level accuracy that can help with interpreting what activities are being done [29,30]. More modern technologies, such as ultrasound or ultra-wideband (UWB) localization, provide higher level accuracies that are more useful for detecting functional activities. Detection of basic activities of daily living (personal hygiene, grooming, dressing, and toileting) and instrumental activities of daily living (managing finances, food preparation, and housekeeping laundry) are critical for effective functional assessment.

Other systematic reviews on indoor localization have focused on the technical measures of accuracy or the range of technologies that could be used to detect activities with localization techniques [31,32]. The focus of this review is to review currently available localization technologies being used for clinical purposes, including the acceptability of devices and measurement of clinical outcomes or diagnoses.

The primary objective of this study is to systematically review the clinical evidence for indoor localization technologies to support in-home monitoring of older adults. Secondary objectives include the following: to review the acceptability of in-home positioning technologies and to summarize the range of localization technologies being developed.

## Methods

### Review Registration and Search Strategy

This systematic review protocol was registered with PROSPERO (ID: CRD42022339845) and follows the methodology of the PRISMA (Preferred Reporting Items for Systematic Reviews and Meta-Analyses) guidelines [33]. The PRISMA checklist can be found in Multimedia Appendix 1.

The search was completed on May 19, 2022, with inclusion criteria displayed in Textbox 1. The search strategy can be found in Multimedia Appendix 2. The search strategy included search terms for the older adult population undergoing in-home positioning or monitoring systems as their intervention. Keywords related to older adults included “Aged,” “Senior,” “Over 65,” or “Aging,” while terms on the setting included their home or house. For the technologies, terms included the purpose of monitoring (“Positioning” or “Localization”) and specific types of technologies, including wireless trackers (Bluetooth, Wi-Fi, UWB, or Zigbee), wearables (accelerometers or gyroscopes), camera, and audio systems. The search did not include comparator groups or outcomes to improve the sensitivity of the search. We included studies that had at least 4 patients to improve the sensitivity of the search. Articles focused on measuring life spaces outside the home (travel to appointments, shopping centers, or recreation centers) were excluded. Studies using wearables were only included if assessed within a home setting.

**Inclusion and exclusion criteria.**

**Abstract inclusion criteria**

- Older adults (65+)
- Monitoring technology
- In-home setting
- Sample size >4 patients

**Full-text inclusion criteria**

- All abstract inclusion criteria
- Positioning system

**Exclusion criteria**

- Care centers (assisted living, long-term care, hospital, etc)
- Conference abstracts
- Reviews and study protocols
- Non-English

### Study Selection, Extraction, and Quality Assessment

MEDLINE, Embase, CINAHL, and Scopus were searched, and articles were deduplicated. Abstract screening, full-text screening, data extraction, and quality appraisal were completed by 2 reviewers: the first author (AC) and 1 of 4 secondary reviewers (SP, BC, LQ, and JC). Reviewers were trained with 10 test abstracts and full-text articles, and then concordance was reviewed. At each stage, interrater agreement was calculated using the κ coefficient calculated by the following formula:



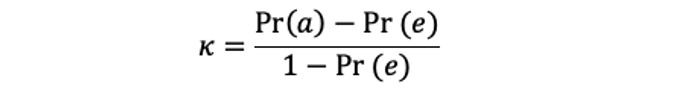



where Pr(*a*) represents the actual observed agreement and Pr(*e*) represents the chance agreement [34]. Disagreements were resolved by having both reviewers reassess articles for 2 additional rounds, and then the article was discussed to reach a consensus.

Data extraction included article demographics (country and year published), study design (clinical, usability, or technical study), population characteristics (age, gender distribution, clinical diagnoses, and comparators), types of localization interventions (wearable or ambient, data transmission, technology readiness level, and data analytics methods), and outcomes (types of activities monitored, clinical assessments and outcomes, acceptability, and reliability). Data were compiled into summary tables, presenting the population, technological intervention, and clinical outcomes of each study.

To assess risk of bias, the JBI checklist for case series critical appraisal tool was used, as we did not expect any high-quality randomized controlled trials related to in-home monitoring [35]. Criteria for appraisal were predetermined: studies with 7 or more “Yes” ratings were considered high quality, studies with 4-6 “Yes” ratings were considered moderate, and studies with fewer than 5 “Yes” ratings were considered low quality. No meta-analysis was planned, as we did not expect to find high-quality quantitative studies that would allow for heterogeneity to be assessed. Instead, the outcomes from each study were presented individually.

Clinical outcomes were summarized in summary statements, with only moderate- or high-quality studies considered. Evidence was summarized as positive if the majority of studies showed positive results, negative if the majority of studies showed negative results, and mixed if neither had a majority.

## Results

### Search Results

**Figure 1:**
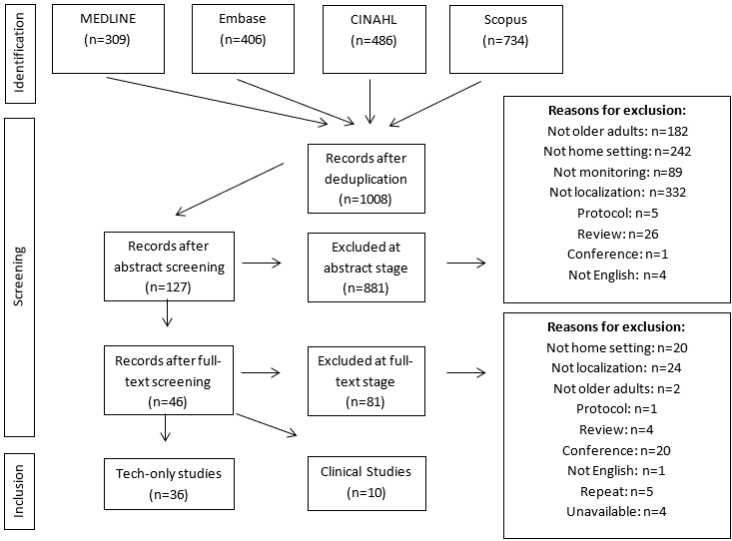
PRISMA (Preferred Reporting Items for Systematic Reviews and Meta-Analyses) flowchart showing included clinical studies (n=10) and technology-only studies (n=39).

During the initial search, 1935 articles were found, with 1008 unique articles after deduplication. After abstract screening, 127 articles remained. After full-text screening, 46 articles were included in the final extractions: 36 technology-focused articles and 10 articles that included relevant patient populations. Agreement between reviewers at the abstract screening stage was 94.9% with a κ of 0.77, and agreement for the full-text screening was 95.8% with a κ of 0.71. Quality assessment agreement was 76% with a κ of 0.51. The PRISMA flowchart in Figure 1 maps out the excluded articles.

### Studies With Clinical Population

Table 1 displays the baseline characteristic for the 10 papers that included relevant patient populations. In total, 7 studies were conducted since 2019. In total, 5 studies were from North America, 3 from Europe, and 2 from Asia. In total, 2 studies were descriptive studies of the technology, 4 studies had a mixed design and qualitative study design, 2 had qualitative study designs, 1 was a mixed study with qualitative and quantitative outcomes, and 1 focused on quantitative outcomes. Only 1 study had more than 25 participants. In total, 8 studies had more female than male participants.

All studies had patient populations that included older adults, although only 7 specifically reported population characteristics. In total, 4 studies included older adults living at home with nonspecific functional challenges, 2 focused on adults with mild cognitive impairment or dementia, and 1 focused on older adults with frailty. Half of the studies (5/10, 50%) were considered low quality, 3 (30%) were considered moderate quality, and 2 (20%) were considered high quality.

Table 2 shows the technologies and localization methods used in the included studies, their setting, and the duration of monitoring. From a technology perspective, 2 used solely an ambient sensor design, 5 combined ambient sensors with wearables, and 3 used wearable-only designs. Ambient sensors included temperature sensors, magnetic door sensors, infrared motion sensors, light switch sensors, pressure detectors, and lidar sensors. Wearables included inertial measurement units (IMUs), electrocardiograms, heart rate meters, and wearable wireless tags (Wi-Fi or Bluetooth low energy). Of the 10 studies, 7 (70%) included technologies of unknown brand or model (3 only used non-branded devices), while 3 (30%) listed the brands of devices used.

Most studies (7/10, 70%) were done in the home setting, with 2 in a home-like laboratory setting and 1 in a laboratory setting. Studies in the laboratory-home involved monitoring sessions lasting between 1 hour and 7 days [28,29,41], while home-based monitoring ranged from 3 weeks to 18 months.

Table 3 displays the outcomes from studies that included patient populations. In total, 7 included technical outcomes, 6 included usability and acceptability outcomes based on patient or clinician surveys or interviews, and 3 included clinical outcomes. Room detection accuracy ranged from 50% to 88% across 3 studies [27,28,30], while 1 study reported failure rates of >15% for motion detectors and servers installed in the home [36]. One study reported linear accuracies of 1.5-2 m using wireless sensor networks within the home [29].

**Table 1:** Baseline characteristics from the included clinical papers.

Author (year)	Country	Design type	Participants, n	Female	Age (years), mean (SD; range)	Population	Category of technology	Quality
Hu et al (2016) [36]	United States	Mixed (qualitative + design)	13	62%	69.2 (NR^a^; 54-85)	Older adults	Ambient	Low
Rahal et al (2008) [28]	Canada	Descriptive	14	71%	50 (NR; 22-73)	Mostly older adults	Ambient	Low
Shin et al (2021) [37]	United States	Mixed (qualitative + design)	23	57%	73 (7.9; 62-89)	Older adults with difficulty conducting activities of daily living	Wearable Ambient Wearable	Moderate
Pais et al (2020) [38]	Switzerland	Qualitative	21	48%	85 (7; 72-96)	Older adults living at home	Ambient Wearable Wearable	High
Lach et al (2019) [27]	United States	Mixed (qualitative + design)	5	100%	86 (5.1; 70-90)	Older adults living alone in home	1. Wearable 2-5. Ambient	Moderate
Hung et al (2021) [29]	Taiwan	Qualitative	8	60%	68 (NR; 64-77)	Adults with mild cognitive impairment or dementia	1. Wearable 2-3. Ambient	Moderate
Rawtaer et al (2020) [39]	Singapore	Mixed (qualitative + quantitative)	49	67%	73 (5.3; NR)	Older adults who are cognitively healthy Older adults with mild cognitive impairment	1-4. Ambient 5. Wearable	High
Montoliu et al (2020) [30]	Spain	Descriptive	17	NR	62.8 (12; 30-79)	Older adults	Wearable	Low
Chen et al (2013) [40]	United States	Mixed (qualitative + design)	4	50%	65 (NR; 46-81)	Range of diagnoses (polio, multiple sclerosis, spinal cord injury)	Wearable	Low
Tegou et al 2019 [41]	Greece	Quantitative	271	56%	76.8 (5.2; NR)	Older adults who are nonfrail Older adults who are prefrail Older adults who are frail	Wearable	Low

^a^NR: not reported.

**Table 2:** Technological setup and technical accuracy of localization devices.

Author (year)	Technology	Brand and model	Localization method	Purpose of monitoring	Setting	Duration	Quality
Hu et al (2016) [36]	Temperature, magnetic door sensor (n=2) Motion sensor (n=12)	Not reported	Motion detection time	Not reported	Home	9-10 weeks	Low
Rahal et al (2008) [28]	Motion sensor (n=10) Tactile carpet (n=18) Light switch (n=8) Door contact (n=48) Pressure detectors (n=1)	Not reported	Motion detection time	Detect walking or preparing sandwich	Home-like laboratory	50 minutes	Low
Shin et al (2021) [37]	Wristband: heart rate, electrodermal activity, triaxial accelerometer (n=1) Lidar sensor (n=1) Camera wearable (n=1)	Empatica E4 FARO Focus S120 Not reported	Camera-based	Functional mobility, BADL^a^, and IADL^b^	Home	18 months	Moderate
Pais et al (2020) [38]	Ambient sensors (not reported) Activity tracker (n=1) Electrocardiogram (n=1)	DomoCare Not reported Preventice BodyGuardian	Passive IR^c^ sensor	BADL (toilet and fridge usage)	Home	12 months	High
Lach et al (2019) [27]	Activity tracker (n=1) Motion detectors (n=3) Bed pressure sensor (n=1) Chair pressure sensor (n=1) Exit sensor (n=1)	CamNtech Motion-Watch 8 Alarm.com BeClose	Motion detection time	Functional mobility, BADL (kitchen, bathroom activity), and sleep quality	Home	3 months	Moderate
Hung et al (2021) [29]	Bluetooth localizer (n=4) Near-field communication scanner voice-guided exercise Voice questionnaire	Not reported	Signal intensity	Cognitive training	Laboratory	5 weeks (intermittent) 60-minute sessions	Moderate
Rawtaer et al (2020) [39]	Passive infrared sensor (n=4) Proximity tags (n=1) Medication box (n=1) Bed sensor (n=1) Pedometer and heart rate meter (n=1)	1-4. Not reported 5. Microsoft Band	Motion detection	Identify mild cognitive impairment or healthy cognition in community-dwelling older adults	Home	2 months	High
Montoliu et al (2020) [30]	Smartwatch (GPS, gyroscope, accelerometer, compass, ambient light sensor; (n=1) Wi-Fi (wireless access point; (n=2) Bluetooth low-energy beacon (n=3) Personal phone (varying)	Sony Smart Watch 3 Not reported iBKS	Signal intensity (received signal strength indicator)	Localization to detect behavioral changes	Home	2 months	Low
Chen et al (2013) [40]	Wi-Fi tag (n=1) Wireless access points (n=3-7) GPS logger	1-2. Ekahau T301A 3. iBlue 860E	Signal intensity (fingerprinting) and GPS	The complete measure of physical activity using various sensors	Home	3-6 weeks	Low
Tegou et al (2019) [41]	Smartphone (n=1) Bluetooth beacons (n=5)	LG Nexus 5x Sensoro	Signal intensity (received signal strength indicator)	Identify frailty in community-dwelling adults	Home	1-7 days	Low

^a^BADL: basic activities of daily living; refers to personal hygiene, grooming, dressing, and toileting.

^b^IADL: instrumental activities of daily living; refers to managing finances, food preparation, and housekeeping laundry.

^c^IR: infrared.

**Table 3:** Outcomes from studies that included patient populations.

Author (year)	Category of technology	Outcomes measured	Technical outcomes	Qualitative outcomes	Clinical outcomes	Quality
Hu et al (2016) [36]	Ambient	Survey: ease of installation, acceptability of sensors, instructions efficiency, device failure rates	Failure rate: <15%: motion detectors and temperature sensors >15%: door sensors, servers, and relays	Ease of use: 2.9 out of 4, high Concerns with devices: 1.6 out of 5, low concerns Instructions efficiency: 80.95%, yes	N/A^a^	Low
Rahal et al (2008) [28]	Ambient	Localization accuracy	Combined: 85% room detection accuracy Accuracy for each device: 88% Not measured 50% 77% Not measured	N/A	N/A	Low
Shin et al (2021) [37]	Ambient and wearable	Patient interviews: adaptive behaviors at home	N/A	For difficult activities, patients most often give up on them or perform slowly Home adaptations are rarely implemented due to cost High fall–risk locations are avoided	N/A	Moderate
Pais et al (2020) [38]	Ambient and wearable	Survey: satisfaction with devices	N/A	Ambient sensors: Older adults: 81.6% positive Caregivers: 80% positive Nurses: 69% positive Wearable sensors: Older adults: 72.2% positive Caregivers: 60% positive Nurses: 49% positive	N/A	High
Lach et al (2019) [27]	Ambient and wearable	Measurement of activity levels and sleep duration Patient interviews: Patient experiences with monitoring	Activity: Self-reported activity and sensor activity correlate Actigraphy did not Sleep: Self-reported: 492 min Actigraphy: 524 min Bed sensor: 435 min	Interview: opinions ranged widely on how noticeable and bothersome ambient sensors were Behaviors sometimes changed due to monitoring presence Compromises to data due to the presence of others in the home is a concern	N/A	Moderate
Hung et al (2021) [29]	Ambient and wearable	Linear localization accuracy Patient survey: system usability Physician survey: availability and quality of system	1.5-2 m in 48×32 m space	Patients: system usability scale: 62.8 SD 11) out of 100 Physicians: cognitive training more targeted and realistic in patients’ home	N/A	Moderate
Rawtaer et al (2020) [39]	Ambient and wearable	Patient interviews: purpose not specified Clinical: comparison between healthy cognition and mild cognitive impairment	N/A	83% positive feedback Improved safety, security, some intrusion where sensors were set up	No behavior difference between healthy cognition and mild cognitive impairment	High
Montoliu et al (2020) [30]	Wearable	Localization accuracy	Room detection accuracy: 50.9%-53.8%	N/A	N/A	Low
Chen et al (2013) [40]	Wearable	Localization accuracy Patient interviews: acceptability of system	Room detection accuracy: 62%-87%	Lightweight tag: little effort is needed when using tags The inclusion of GPS is helpful	N/A	Low
Tegou et al (2019) [41]	Wearable	Clinical: identify frailty in community dwelling adults	N/A	N/A	Accuracy in classifying frailty: 80%-87%	Low

^a^N/A: not available.

For studies that included usability and acceptability outcomes, surveys from 3 studies [29,36,38] showed positive results. One study focused on ease of setup of a smart home in a box design and found high ease of use, few concerns with devices, and highly efficient instructions [36]. Another found the highest satisfaction among older adults, followed by caregivers, and the lowest satisfaction with nursing staff [38]. One study found an average system usability scale score of 62.8, indicating below average usability [29]. Interview results from 4 studies [27,37,39,40] found improved safety and security with devices, but there was some perceived physical intrusiveness to ambient devices [39], and some patients changed their behavior due to monitoring [27]. One study found a tag-based system was highly acceptable [40].

Lastly, regarding clinical outcomes, 1 study provided qualitative observations on why patients behaved in certain ways in their home, finding certain activities are performed slower and some areas in the home are avoided, including staircases to avoid falls, depending on their functional level [37]. One study found no difference in behaviors between residents with mild cognitive impairment and those who were cognitively healthy, based on continuous monitoring of sleep and identifying frequency of forgetting to do activities [39], while another study was able to classify patients as frail, prefrail, or nonfrail with 80% to 87% accuracy using machine learning algorithms from Bluetooth-based wearable localization, being monitored for 1-7 days continuously in their own home while doing their own typical activities [41].

### Studies on Technology Validation

The primary objective of this systematic review was to review the clinical evidence for in-home localization technologies to support in-home monitoring of older adults. We found 36 articles that reported that their technology would be used for localization of clinical populations. Table 4 is a summary of the characteristics of studies focused on developing and evaluating in-home localization technologies for older adults.

Studies on ambient sensors were from North America (3/6 studies, 50%), wireless tags were most studied in Europe (6/6, 100% for Bluetooth or Wi-Fi and 5/7, 71% for other tags), and wireless tags alongside IMUs were solely studied in Asia (8/8, 100%). The majority (25/36, 69%) of studies were from after 2016. The stated purpose of monitoring was for older adults in a general sense in 27 (75%) out of 36 studies, while older adults with chronic diseases or disabilities were specified in 9 (25%) studies. The purpose of monitoring was mostly for health and safety monitoring (21/36, 58%).

The most common localization mode was to measure signal strength (23/36, 64%), followed by time-based localization (8/36, 22%), which calculates the time that it takes for a signal to travel from a tag to a reference point, and the least common was proximity sensing (5/36, 14%). Received signal strength involves estimating the distance between wearables and reference points based on the strength of the wireless signal. Localization accuracy was most reported as a linear distance (23/36, 64%), followed by classification of activities (13/36, 36%), room or area detection accuracy (6/36, 17%), and lastly accuracy in detecting multiple people in a space (5/36, 14%).

Table 5 summarizes the accuracies of different technologies, organized according to the method of localization and the type of accuracy reporting. Ambient sensors included infrared sensors, radiofrequency transceivers, and video feedback. Devices were primarily used for detecting people passing through spaces, with accuracies of 79% to 98% in differentiating people, and 92% accuracy in detecting presence in a room.

**Table 4:** Characteristics of studies focused on monitoring technologies.

Category and subcategory		Ambient sensor (video, infrared, magnetic, or pressure), n	Bluetooth or Wi-Fi, n	Localizer tag (RFID^a^, UWB^b^, Zigbee, or GPS), n	IMU^c^ and localizer, n	Other, n	Total, n
Articles		6	6	7	8	9	36
Continent							
	Europe	1	6	5	0	1	13
	Asia	1	0	1	8	3	13
	North America	3	0	0	0	5	8
	Oceania	1	0	1	0	0	2
Year							
	Before 2010	0	1	0	0	1	2
	2010-2016	4	1	0	3	1	9
	2016-2022	2	4	7	5	7	25
Target audience							
	Older adults	4	4	6	7	6	27
	Older adults with chronic disease	2	0	1	1	3	7
	Older adults with disabilities	0	2	0	0	0	2
Purpose of monitoring							
	Indoor localization	3	0	1	1	0	5
	Activity detection	1	0	0	3	2	6
	Health or safety monitoring	2	3	5	4	7	21
	Self-care	0	3	1	0	0	4
Localization mode							
	Signal strength	2	6	5	7	3	23
	Proximity sensing	4	1	0	0	3	5
	Time-based localization	0	0	2	1	2	8
Accuracy reporting							
	Distance	2	3	6	6	6	23
	Activity classification	2	0	2	5	4	13
	Room or floor or area detection	1	3	0	2	0	6
	Multiple tag and person detection	3	0	1	0	1	5

^a^RFID: radio frequency identification.

^b^UWB: ultra-wideband.

^c^IMU: inertial motion unit.

**Table 5:** Accuracy reporting from localization technologies.

Localization technologies			Distance		Activity classification		Room or floor or area detection		Multiple tag and person detection
Ambient sensors (video, IR^a^, magnetic, or pressure; 6 studies)									
	Studies, n/N (%)	1/2 (50)		2/2 (100)		1/1 (100)		3/3 (100)	
	Accuracy	Thermopile sensor: 12-65 cm [11] IR sensors: not reported [12]		RF^b^ transceiver [13]: walking: 97%, standing: 95% Video [14]: sensor placement optimization: 98%		RF transceiver [13]: room detection: 92%		RF transceiver [13]: >1 person: 79%-90% IR and RF transceiver [15]: 2 male individuals: 83% vs 1 male and 1 female individual: 98% IR doorway sensor [16]: 1 person: 89%, 2 people: 81%	
Bluetooth or Wi-Fi (6 studies)									
	Studies, n/N (%)	4/4 (100)		—^c^		3/3 (100)		—	
	Accuracy	Wireless sensor network: <250 cm [17] Bluetooth: 60-300 cm [18], 70-240 cm [19], 86 cm [20]		—		Bluetooth [18]: area accuracy (1 m×1 m): 95%, room detection accuracy [21]: 75%-84% Wi-Fi [22]: room detection accuracy: 70%-87%		—	
Localizer tag (RFID^d^, UWB^e^, Zigbee, or GPS; 7 studies)									
	Studies, n/N (%)	5/6 (83)		2/2 (100)		1/1 (100)		1/1 (100)	
	Accuracy	RFID: 17 cm [23], not reported [24] UWB: 5 cm [25], 5-20 cm [26] Zigbee [27]: 92 cm UWB+BLE^f^ [28]: 23-100 cm		UWB [26]: fall detection: sensitivity 99%, specificity 98% RFID [29]: object identification 88%		RFID [23]: area accuracy (1.1 m×1.2 m): 90%		RFID [29]: multitag sensitivity: 76%-90%	
IMU^g^ and localizer (8 studies)									
	Studies, n/N (%)	6/6 (100)		5/5 (75)		2/2 (100)		—	
	Accuracy	IMU+UWB: 7.6 cm [30] IMU+RFID: 10-40 cm in 3.6×2.8 m [31], <50 cm [32] IMU+Zigbee: 120 cm in 11 m×5.75 m [33], 83-189 cm [34] IMU+BLE: 47 cm [35]		IMU+Zigbee: fall detection: 89% [33], unspecified activity: 100% [41] IMU+RFID: posture recognition: 100% [32] IMU+BLE: step count within 1 step/minute [35] IMU+BLE: activity classification: 95% [36]		IMU+Zigbee: area accuracy (2 m×2 m): 90% [41] IMU+BLE: room detection accuracy 86.6% [36]		—	
Others (9 studies)									
	Studies, n/N (%)	5/6 (83)		3/4 (75)		—		0/1 (0)	
	Accuracy	Triboelectric tracker [38]: at 1.5 m, 20-30 cm Unspecified doorway sensors: distance traveled error: 10.5%-24% [40] Android location-based service: not reported [39] Ultrasound+RF: 11 cm [37] Floor vibration sensor: 24-61 cm [42] BLE+Acoustic+Light Fidelity: 20 cm [43]		IMU+Mic+Wi-Fi: ADL recognition: 92-99% [44] Unspecified doorway sensors: activity detection: 92% [40] Ultrasound+RF: gait speed error: 91% [37], distance walked: 92% Floor vibration sensor: footstep detection: 95%-99% [42] Ambient+Scales+IMU: not reported [45]		—		IR+Pressure Pad+RF transceiver: not reported [46]	

^a^IR: infrared.

^b^RF: radio frequency.

^c^Not applicable.

^d^RFID: radio frequency identification.

^e^UWB: ultra-wideband.

^f^BLE: Bluetooth low energy.

^g^IMU: inertial measurement unit.

Bluetooth and Wi-Fi technologies can be used with either smartphones or individual tags, reducing the need for extra equipment for localization when compared to stand-alone tags. Accuracies ranged from 70-250 cm, with room detection accuracies of 70% to 87%.

Localizer tags include radio frequency identification (RFID), UWB, Zigbee, and GPS tags. Linear accuracies were superior to Bluetooth or Wi-Fi, ranging from 5 to 100 cm, with area accuracies of 90%. Tags were also used for fall detection and object detection.

Combining localizers with IMUs allowed for a combination of activity classification and localization. Accuracies ranged from 7.6 to 189 cm across 4 modalities (UWB at 7.6 cm, RFID at 10-40 cm, Zigbee at 83-189 cm, and Bluetooth low energy at 47 cm), while activity classification ranged from 89% to 100%, although reporting was not always clear on what activities were being classified. Area classification accuracies were between 86% and 90%.

Lastly, with unique technologies, including sound-based technology, GPS, vibration sensors, pressure pads, and triboelectric sensors, accuracies ranged from 20 to 30 cm with activity recognition at 92% to 99%.

### Summary Statements on Clinical Evidence for Localization

From the 5 moderate- to high-quality clinical studies, 4 studies reported on acceptability of in-home localization systems. Results were mixed, with 2 high-quality studies indicating positive acceptability [38,39], 1 finding below average usability [29], and 1 finding a range of concerns over device obtrusiveness [27].

Two studies reported on clinical outcomes from in-home localization systems. One high-quality study showed no difference in behaviors in older adults with healthy cognition compared with those with mild cognitive impairment [39], and 1 moderate-quality study detected adaptive behaviors at home because of limitations to patient function [37].

## Discussion

### Principal Findings

This systematic review focused on the usage of localization methods to monitor older adults in their homes for any clinical application. While the primary objective was to evaluate the clinical evidence for localization technologies, a survey of technologies for in-home localization was also undertaken to understand upcoming technologies for localization.

Clinical utility of localization was mixed in this study. In the study by Rawtaer et al [39], cognitively healthy older adults (21 participants) and older adults with mild cognitive impairment (28 participants) were monitored and compared over 2 months using a custom set of motion sensors, proximity tags, a bed sensor, and wearables to capture sleep; activity levels; and forgetfulness regarding medications, keys, or wallets. Among typical activities, there was no difference in behaviors [39]. A second study, examining frailty, used in-home localization to detect frailty by measuring number of transitions, speed of transitions, and statistical features through machine learning algorithms, finding a classification accuracy of 82% to 85% when using random forest plots [41]. The model can be used in the future to detect frailty in the general population. The clinical evidence for using localization technology to support care of older adults is currently limited.

From an acceptability perspective, results were moderately positive [27,29,36,38,39]. Pais et al [38] discovered that ambient sensors garner greater acceptance compared to wearables. Moreover, they noted that older adults and caregivers exhibit higher acceptance levels toward both technologies in contrast to nurses. This trend could be attributed to the necessity for monitoring daily performance issues among older adults and their families. The acceptability of home monitoring has been thoroughly studied previously, finding that the trade-offs are critical to consider when developing these technologies [42-45]. These findings align with the present systematic review, with obtrusiveness being a major detractor for these localization technologies balanced by improved safety and security.

Common technologies for localization include ambient sensors; Bluetooth- or Wi-Fi–received signal strength; localizer tags using RFID, UWB, Zigbee, GPS; or IMUs with localizers. This review also found unique localization devices, including triboelectric trackers, ultrasound, floor vibration sensors, and pressure pads. Highest linear accuracies were found with UWB technologies at 5-20 cm compared with greater than 50 cm for most other technologies. UWB uses time-based localization, which involves measuring the time it takes for a signal to travel from a tag to a reference point and then trilateralizing the signal. Room detection accuracies were comparable across technologies, ranging from 75% to 92% using Bluetooth, Wi-Fi, RFID, radio frequency transceivers, or Zigbee with IMUs.

The current literature is limited as it focuses primarily on technical measures of accuracy. The shift needs to be made toward localization for activity identification that can then be used as evidence to provide an intervention. UWB positioning has the potential to make the shift from where a patient is in the home at a room level to a furniture level that can then allow identification of activities. Further exploration and development of algorithms to automatically detect activities are required before broader clinical usage.

### Comparison to the Literature

This systematic review fills an important gap by including clinical results, user acceptability, and technological aspects of evaluating localization devices to support older adults to age in place. There remains little evidence for their usage for older adults, a finding that is supported by other systematic reviews. Lenouvel et al [31] reviewed sensors to measure and support activities of daily living for older adults in 2019. While they did not focus on localization, they found that passive and video sensor networks were used to assess activities of daily living across 13 studies out of their search of 10,782 studies, finding that sensors could detect changes in activity patterns but reported no clinical outcomes and that only 1 study assessed the acceptability of devices.

Another systematic review published in 2018 focused solely on technological aspects of human activity recognition supported with indoor localization. Cerón and López [32] described common localization technologies and data fusion methods, reporting accuracy of activity detection and localization accuracies without consideration for age of participants. Human activity recognition accuracy ranged from 72% to 99% across 27 studies, although the exact types of activities were not reported. Localization accuracies ranged from 0.8 to 7 m, depending on the type of technology, although the type of technology was not reported in the review. These values are comparable to this systematic review.

### Strengths of This Study and Recommendations for Future Studies

This systematic review had a strong search strategy, covering the major databases and having 2 reviewers screen, extract, and assess the quality of studies. Agreement between reviewers was high across screeners. The JBI quality assessment tool was used with a lower agreement with a κ of 0.51. Agreement was low due to inadequate training for the 10 clinical articles, a lack of specific definitions of how much clinical information was adequate for the study, and how follow-up was defined in the article. Each study was discussed between reviewers according to a standardized definition for the final results of this study.

While the methodology of this review was strong, the findings were not. There is limited clinical evidence for using localization to support monitoring older adults. It was surprising that there were also few studies that evaluated the acceptability of monitoring technologies. The quality of evidence also needs to be improved, with most studies having fewer than 25 participants with a case study design and the quality of studies being mixed.

Still, existing studies on acceptability of localization technologies form a strong basis for further development. Future studies should be located within the homes of participants, with sample sizes greater than 25 to demonstrate scalability and particular use cases in a broad range of home settings. From a study-design perspective, home monitoring as an intervention is a complex intervention that is challenging to capture in a randomized controlled trial. The recent guidelines by the Medical Research Council and National Institute for Health Research in the United Kingdom provide a new framework for assessment that includes considering the context, stakeholders, economics, and uncertainties in an intervention, grounding it in appropriate theory, and iterating to refine the intervention [46]. Nonrandomized designs, hybrid effectiveness-implementation designs, or n-of-1 trials may be more feasible.

Details around patient populations were scarce. Greater detail in medical histories, functional ability, and practical aspects (social supports, living spaces) need to be provided to generate profiles for how monitoring interventions have helped specific residents. A standard battery of activities of daily living needs to be established to allow accuracy in identifying and assessing activities of daily living to be comparable across studies in the range of indoor spaces being localized and the diversity of impairments common to older adults. There needs to be clearer reporting of the spaces being monitored, accuracy of devices, and types of activities of daily living being monitored to allow comparability. Lastly, study outcomes need to be shaped to demonstrate how monitoring technologies lead to clinically and personally relevant interventions that support aging in place. The 2 studies that looked at clinical outcomes in this systematic review focused on detecting dementia or classifying frailty. Perhaps the more important question is how minimally invasive interventions can be used to either prevent decline or intervene to support residents who are having greater challenges doing self-care activities.

The utility of localization techniques for health care is still untapped. While some initial work on detecting cognitive decline and frailty in the home setting has been documented in this review, further development and clinical evaluation of these technologies to determine potential use cases still needs to be undertaken. Development of these technologies requires a multipronged approach that combines understanding the limits of the technology, including the cost, the clinical applicability of localization for health management, and the acceptability of monitoring to enhance wellness. Technologies such as Bluetooth, Wi-Fi, and IMUs are already well established in the market for various quality of life use cases but not for health care.

Localization could be a powerful supporting tool for managing challenges with cognition, with interventions that take into account a user’s living patterns and reminders that are tailored to the home environment. Of upcoming technologies, UWB may be the most exciting, offering much higher accuracies than ambient sensors and wireless technologies such as Wi-Fi and Bluetooth. Cognition, mental health, and frailty could be more accurately measured longitudinally, rather than relying on snapshot clinical assessment tools when combined with collecting information on self-care and in-home activity levels. There is great potential for localization technologies to support wellness in the home.

### Conclusions

There is no evidence for the usage of in-home localization technologies for any clinical outcomes and mixed evidence for the acceptability of localization technologies among older adults. However, there is a wide range of technologies available that have promising technical accuracy. The technology is ripe for monitoring devices to be tested clinically, providing data that can detect changes in cognition or frailty and drive interventions. Further study on the acceptability of these devices is also warranted to determine the least obtrusive and easier to use modalities that can bring the most benefit for older adults.

## Appendix

Multimedia Appendix 1 PRISMA (Preferred Reporting Items for Systematic Reviews and Meta-Analyses) checklist.

Multimedia Appendix 2 Detailed search strategy for the systematic review.

## Abbreviations

IMU: inertial measurement unit
PRISMA: Preferred Reporting Items for Systematic Reviews and Meta-Analyses
RFID: radio frequency identification
UWB: ultra-wideband
