# Supplementary material for: In-Home Positioning for Remote Home Health Monitoring in Older Adults: Systematic Review
Source: JMIR Aging. 2024 Dec 2;7:e57320. doi: 10.2196/57320 (PMC11661402; doi:10.2196/57320)
Supplement: Multimedia Appendix 2 [file aging_v7i1e57320_app2.pdf]

Medline and Embase

1	Geriatric Assessment/ or Geriatrics/
2	exp aged/
3	("over 65" or "65 and over" or "65 or over" or "65+ years" or frail* or retired or sarcopeni* or elder* or old* or old age or centenarian* or nonagenarian* or octogenarian* or septuagenarian* or aging or (senior* not ((high school or university or college) adj3 senior*)) or gerontolog* or geriatric* or mature adult* or mature patient* or mature individual* or aged adult* or aged patient* or aged individual* or aged population* or (older adj2 (people or person* or client* or adult* or patient* or individual* or population* or women or men)) or dementia or parkinson* or stroke).mp.
4	((house* or home or homes or homebound) adj8 (positioning or positioner or localiz* or localis*)).mp.
5	((Indoor adj8 (track* or position* or localiz* or localis*)) not indoor track).mp.
6	(((positioning system* not global positioning system*) or location tracking or fitness track* or position tracking or position* monitoring or monitor* position* or position* measurement*) and (house* or home or homes or homebound or indoor*)).mp.
7	((home or homes or homebound or house*) and ((wireless or infrared or IR or RFID or Radio-frequency identification or RF or radio-frequency or zigbee or Bluetooth or BLE or ultra-wide-band or UWB or Wi-fi or camera* or video* or "time of flight" or wearable* or "mobile device*" or mhealth or smartphone* or inertia* or imu or imus or accelerometer or gyroscope or "motion sensor*" or wan or iot or internet-of-things or "internet of things") adj8 (track* or position* or localiz* or localis*))).mp.
8	((indoor* or house* or home or homes or homebound) adj10 (track* activit* or activit* track* or track* movement* or movement tracking)).mp.
9	local positioning system*.mp.
10	(global positioning system* adj10 (indoor* or house* or home or homes or homebound)).mp.
11	1 or 2 or 3
12	4 or 5 or 6 or 7 or 8 or 9 or 10
13	11 and 12

CINAHL

#	Query
S17	S15 AND S16
S16	S7 OR S8 OR S9 OR S10 OR S11 OR S12 OR S13 OR S14
S15	S1 OR S2 OR S3 OR S4 OR S5 OR S6
S14	(global positioning system* N10 (indoor* or house* or home or homes or homebound))
S13	local positioning system*
S12	((indoor* or house* or home or homes or homebound) N10 (track* activit* or activit* track* or track* movement* or movement tracking))
S11	((home or homes or homebound or house*) and ((wireless or infrared or "IR" or RFID or Radio-frequency identification or "RF" or radio-frequency or zigbee or Bluetooth or BLE or ultra-wide-band or UWB or Wi-fi or camera* or video* or "time of flight" or wearable* or "mobile device*" or mhealth or smartphone* or inertia* or imu or imus or accelerometer or gyroscope or "motion sensor*" or wan or iot or internet-of-things or "internet of things") N8 (track* or position* or localiz* or localis*))).mp.
S10	((indoor* or house* or home or homes or homebound) N10 (track* activit* or activit* track* or track* movement* or movement tracking))
S9	(((positioning system* not global positioning system*) or location tracking or fitness track* or position tracking or position* monitoring or monitor* position* or position* measurement*) and (house* or home or homes or homebound or indoor*))
S8	((Indoor N8 (track* or position* or localiz* or localis*)) not indoor track)
S7	((house* or home or homes or homebound) N8 (positioning or positioner or localiz* or localis*))
S6	(MH "Geriatric Assessment+") OR (MH "Rehabilitation, Geriatric") OR (MH "Geriatric Functional Assessment")
S5	(MH "Aged+")
S4	("over 65" or "65 and over" or "65 or over" or "65+ years" or frail* or retired or sarcopeni* or elder* or old*or old age or centenarian* or nonagenarian* or octogenarian* or septuagenarian* or aging or (senior* not((high school or university or college) N3 senior*)) or gerontolog* or geriatric* or mature adult* or mature patient* or mature individual* or aged adult* or aged patient* or aged individual* or aged population* or (older N2 (people or person* or client* or adult* or patient* or individual* or population* or women or men)) or dementia or parkinson* or stroke)
S3	geriatric assessment
S2	geriatric
S1	aged

Scopus
19 TITLE-ABS-KEY (17 AND 18)

18 TITLE-ABS-KEY (8 OR 9 OR 10 OR 11 OR 12 OR 13 OR 14 OR 15)

17 TITLE-ABS-KEY (2 OR 4 OR 5 OR 6 OR 7 OR 8)
15 TITLE-ABS-KEY ("global positioning system*" W/10 (indoor* OR house* OR home OR homes OR homebound))

14 TITLE-ABS-KEY (local AND positioning AND system*)

13 TITLE-ABS-KEY ((indoor* OR house* OR home OR homes OR homebound) W/10 ("track* activit*" OR "activit* track*" OR "track* movement*" OR "movement tracking"))

12 TITLE-ABS-KEY (((home OR homes OR homebound OR house*) AND ((wireless OR infrared OR ir OR rfid OR "Radio-frequency identification" OR rf OR "radio-frequency" OR zigbee OR bluetooth OR ble OR ultra-wide-band OR uwb OR wi-fi OR camera* OR video* OR "time of flight" OR wearable* OR "mobile device*" OR mhealth OR smartphone* OR inertia* OR imu OR imus OR accelerometer OR gyroscope OR "motion sensor*" OR wan OR iot OR internet-of-things OR "internet of things") W/8 (track* OR position* OR localiz* OR localis*))))

11 TITLE-ABS-KEY ((((positioning AND system* AND NOT "global positioning system*") OR "location tracking" OR "fitness track*" OR "position tracking" OR "position* monitoring" OR "monitor* position*" OR "position* measurement*") AND (house* OR home OR homes OR homebound OR indoor*)))

10 TITLE-ABS-KEY (indoor W/8 (track* OR position* OR localiz* OR localis*) AND NOT "indoor track")

9 TITLE-ABS-KEY ((house* OR home OR homes OR homebound) W/8 (positioning OR positioner OR localiz* OR localis*))
8 TITLE-ABS-KEY (senior* AND NOT ("high school" OR university OR college) W/3 senior*)
7 TITLE-ABS-KEY ("over 65" OR "65 and over" OR "65 or over" OR "65 years" OR frail* OR retired OR sarcopeni* OR elder* OR old* OR "old age" OR centenarian* OR nonagenarian* OR octogenarian* OR septuagenarian* OR aging)

6 TITLE-ABS-KEY (older (W/2 (people OR person* OR client* OR adult* OR patient* OR individual* OR population* OR women OR men) OR dementia OR parkinson* OR stroke))
5 TITLE-ABS-KEY "over 65" OR "65 and over" OR "65 or over" OR "65+ years" OR frail* OR retired OR sarcopeni* OR elder* OR old* OR "old age" OR centenarian* OR nonagenarian* OR octogenarian* OR septuagenarian* OR aging OR gerontolog* OR geriatric* OR "mature adult*" OR "mature patient*" OR "mature individual*" OR aged AND adult* OR "aged patient*" OR "aged individual*" OR "aged population*" OR ("older" W/2 (people OR person* OR client* OR adult* OR patient* OR individual* OR population* OR women OR men)) OR

dementia OR parkinson* OR stroke OR (senior* AND NOT (("high school" OR university OR college) W/3 senior*))
4 TITLE-ABS-KEY ("over 65" OR "65 and over" OR "65 or over" OR "65+ years" OR frail* OR retired OR sarcopeni* OR elder* OR old* OR old AND age OR centenarian* OR nonagenarian* OR octogenarian* OR septuagenarian* OR aging)
2 TITLE-ABS-KEY (aged OR geriatric OR "geriatric assessment")
